# Role of Phase Nanosegregation in the Photoluminescence Spectra of Halide Perovskites

**DOI:** 10.1021/acs.jpclett.1c03378

**Published:** 2021-11-26

**Authors:** Alessia Di Vito, Alessandro Pecchia, Matthias Auf der Maur, Valerio Campanari, Faustino Martelli, Aldo Di Carlo

**Affiliations:** † University of Rome “Tor Vergata”, Via del Politecnico 1, 00133, Rome, Italy; ‡ CNR-ISMN, Via Salaria km 29,300, 00014 Monterotondo Stazione, Rome, Italy; § CNR-IMM, Via del Fosso del Cavaliere, 00133, Rome, Italy; ∥ LASE, Laboratory of Advanced Solar Energy, National University of Science and Technology “MISiS”, Leninsky prospect 4, 119049, Moscow, Russia

## Abstract

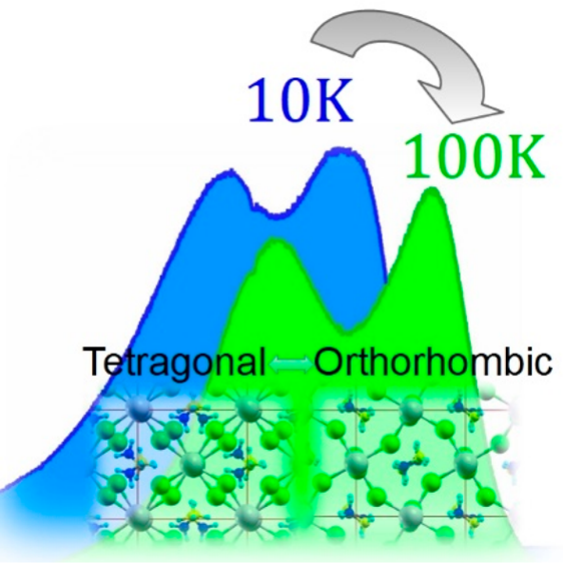

The study of MAPbI_3_ phase transitions based on temperature-dependent optical spectroscopy has recently gained a huge attention. Photoluminescence (PL) investigations of the tetragonal–orthorhombic transition suggest that tetragonal nanodomains are present below the transition temperature and signatures associated with tetragonal segregations are observed. We have studied the impact of phase nanosegregation across the orthorhombic–tetragonal phase transition of MAPbI_3_ on the system’s properties employing a tight binding (TB) approach. The particle swarm optimization has been used to obtain a consistent set of TB parameters, where the target properties of the system have been derived by first-principles calculations. The theoretical results have been compared with the measured PL spectra for a temperature range going from 10 to 100 K. Our model effectively captures the carriers’ localization phenomenon induced by the presence of residual tetragonal nanodomains and demonstrates that the assumption of phase nanosegregation can explain the low-energy features in the PL spectra of MAPbI_3_.

The major advance in the field of photovoltaics of the past decade has been represented by hybrid halide perovskites. These materials have general formula AMX_3_, where A is cesium, methylammonium or formamidinium, M is tin or lead, and X is chlorine, bromine, or iodine. Hybrid lead halide perovskites have been initially studied in dye-sensitized solar cells,^1^ and in a few years, they have shown a stately efficiency improvement.^2−8^ Perovskite materials have been successfully implemented also in a variety of optoelectronic applications, such as light-emitting devices^9^ and lasing.^10^ Their success is due not only to their set of impressive electronic properties^2,11−16^ but also to the possibility of using low-cost techniques for their synthesis and deposition, such as solution-based methods.^5^

In spite of this success, there are still many open questions on the basic properties of this class of materials. In particular, hybrid perovskites show signs of phase transitions that take place in a temperature range of interest for technological applications. Specifically, for the prototypical methylammonium lead iodide perovskite (MAPbI_3_), X-ray diffraction analysis^16,17^ and calorimetric measurements^18,19^ reported the occurrence of two phase transitions at 162 and 327 K, which are known to influence its optical properties.^20−23^ Poglitsch and Weber assigned the low-temperature transition to a change from an orthorhombic-to-tetragonal crystalline structure, and the high-temperature one to a change from a tetragonal-to-cubic structure.^17^ Different behaviors have been reported for the orthorhombic-to-tetragonal phase transition of single crystal and thin film perovskites. Concerning thin films, the transition is observed at lower temperatures, and it involves a wider temperature range,^24^ depending on aspects such as strain, disorder, or polycrystallinity. In this case, the presence of tetragonal phase far below 160 K and down to 10 K was demonstrated.^20,25−27^ In the case of single crystals, scattering studies report that the phase transition occurs in a small temperature range.^28,29^

However, recent studies based on temperature-dependent optical spectroscopy exhibit the presence of different peaks and shoulders in the photoluminescence (PL) spectra of MAPbI_3_ single crystals. Such PL features are observed at lower temperatures with respect to the orthorhombic-tetragonal phase transition temperature.^20,28,30^ Schötz et al. investigated these additional peaks for pure tetragonal and orthorhombic phases.^31^ Furthermore, they analyzed the tetragonal–orthorhombic phase transition of a MAPbI_3_ single crystal employing temperature-dependent PL spectroscopy.^32^ They found that 0.015% of residual tetragonal phase is observed at 150 K. Specifically, they estimated that the size of the tetragonal phase segregations is approximately 7–15 nm at 120 K.

Thus, it is paramount for the success of perovskite materials to understand the relationship between the crystal structure of perovskite and its electronic and optical properties. In theoretical studies related to halide perovskites, the MAPbI_3_ system has been widely used as prototypical model.^33^ Several challenges have to be tackled in order to successfully model the optical properties of such phase segregated nanodomains. The first problem is related to modeling of the individual bulk structures of orthorhombic and tetragonal phases and the matching of the two crystalline structures in the polycrystalline system. A clear issue in this context is that density functional calculations are out of reach for systems with hundreds of thousands of atoms; hence, accurate empirical methods are needed. The second related problem is how to obtain reliable electronic states of the nanodomains. A connected issue is how to set the correct band alignment between the tetragonal and the orthorhombic phases.

Our model is based on several ingredients, pivoting around a tight binding (TB) calculation of the electronic structure and optical transitions. TB was chosen because it intrinsically takes into account for the atomic details of the structures, in contrast to envelop function methods (e.g., *k*·*p*). However, TB requires a careful construction of the atomic model at the orthorhombic/tetragonal interfaces in order to avoid artifacts. In particular, the TB parameters needs to be exportable across different phases and the effect of bond distortion at the interfaces needs to be taken into account. This goal has been achieved by constructing a fully consistent parametrization, able to reproduce the density functional theory (DFT) bulk band structures of orthorhombic, tetragonal, and pseudocubic phases of halide perovskites. The cubic phase was added in order to increase the amount of constrains in the fitting procedure and obtain a parametrization valid throughout the whole temperature scale. This approach, together with proper cross checks on strained crystals allowed to obtain a consistent set, valid also at the interfaces, where bonds are significantly distorted.

In order to construct atomistic models of the tetragonal nanocrystals embedded in the orthorhombic matrix, we have developed a new scheme within the TiberCAD atomistic structure generator,^34^ now able to fill different spatial regions with different atomic crystalline structures and gluing them together with a correct bond-map. This approach is rather in contrast to the usual construction of pseudomorphic crystalline structures that share the same unit cells (like in III–V heterostructures).

The band-edge discontinuity at the interfaces is another parameter needed in TB calculations. Since a reliable value for the interfaces of interest in the present work is not directly available in the literature, we have computed it using a DFT approach. We should remark that DFT, being a ground-state theory, can only provide the valence-band (VB) discontinuity, whereas the energy gaps are fixed from experimental values and the conduction band discontinuity follows as a consequence.

Here, we study the role of phase nanosegregation across the orthorhombic-tetragonal phase transition of MAPbI_3_ on the electronic and optical properties of the system. We simulate the presence of tetragonal nanodomains within the orthorhombic phase. The spatial localization of carriers, induced by the increasing dimension of the tetragonal domains, and its impact on the emission properties of perovskite are analyzed. The theoretical results are compared with the measured PL spectra for a temperature range going from 10 to 100 K.

In order to describe the atomic scale features of the system, we employed the TB module of the TiberCAD software package.^34^ Since the TB method is not designed to yield the optimized crystal structure, a DFT calculation has been employed to relax the atomic positions. DFT simulations have been performed at the GGA level of approximation using the Quantum Espresso package^35^ with scalar-relativistic PBE pseudopotentials.^36^ The kinetic energy cutoff has been set to 25 Ry for wave functions and 200 Ry for charge density and potential, and we used a 4 × 4 × 4 *k*-points grid for reciprocal space integration. It has been demonstrated that the electronic structure of MAPbI_3_ can be reproduced with sufficient accuracy employing the minimal sp^3^ TB basis set.^37^ Thus, we followed the prescription of Boyer-Richard et al.^37^ for the choice of TB basis. Furthermore, DFT calculations, investigating the specific contribution of each atomic species to the electronic states around the band gap, highlight that the weight associated with the MA cation is not relevant.^38^ Based on the mentioned results, our TB model includes only the Pb–I cage and does not include the organic cation. In order to derive the theoretical optical spectra, the momentum matrix elements associated with each optical transition are needed. These are calculated from the electron and hole states evaluated with the TB approach. We considered the first 12 electron and the first 12 hole states, and the *G*, *X*, *Z*, *M*, *R*, and *A* high-symmetry points of the Brillouin zone for reciprocal space integration. The phase-segregated system is modeled placing a tetragonal domain, with nanometric dimensions, at the center of an orthorhombic box, with a fixed side length of 25 nm. According to the estimate presented in the study of Schötz et al.,^32^ we considered tetragonal nanodomains with dimensions of 5, 9, and 14 nm.

The TB Hamiltonian matrix elements (HME) are derived as fitting parameters able to reproduce the target DFT band structure. In order to optimize the fitting procedure, we used the particle swarm optimization (PSO) algorithm.^40^ Note that the optimization of the TB parameters is performed taking into account the band structure of all the three MAPbI_3_ phases simultaneously. Moreover, the experimental band gap value has been taken into account as target feature, adjusting the DFT energy gap to the measured one.^33^ This is discussed in detail in Section I of the Supporting Information. In parts a and b of Figure 1, the TB band structures of, respectively, orthorhombic and tetragonal MAPbI_3_ obtained after the PSO are shown. The related energy gap values are listed in Table 1. Our results are compared with the measurements and GW calculations found in the reference study of Quarti et al.,^33^ also reported in the table. The orthorhombic energy gap value derived by our TB model is consistent with the reference GW calculation and both approaches overestimate the experimental result. Nevertheless, the energy gap difference between tetragonal and orthorhombic MAPbI_3_ obtained with TB is in agreement with measurements. Since we are interested in simulating the presence of tetragonal domains below the phase transition temperature and its effect on the emission properties of MAPbI_3_, a consistent energy gap difference between the two crystals is paramount to properly describe the confinement of carriers and the related spatial localization. For completeness, the effective masses for holes and electrons obtained by parabolic fitting of the TB valence and conduction band edge for tetragonal MAPbI_3_ along the directions Γ → *X* and Γ → *Z* are reported in Table 2. Our results are compared with the theoretical values obtained by Umari et al.^39^

**Figure 1:**
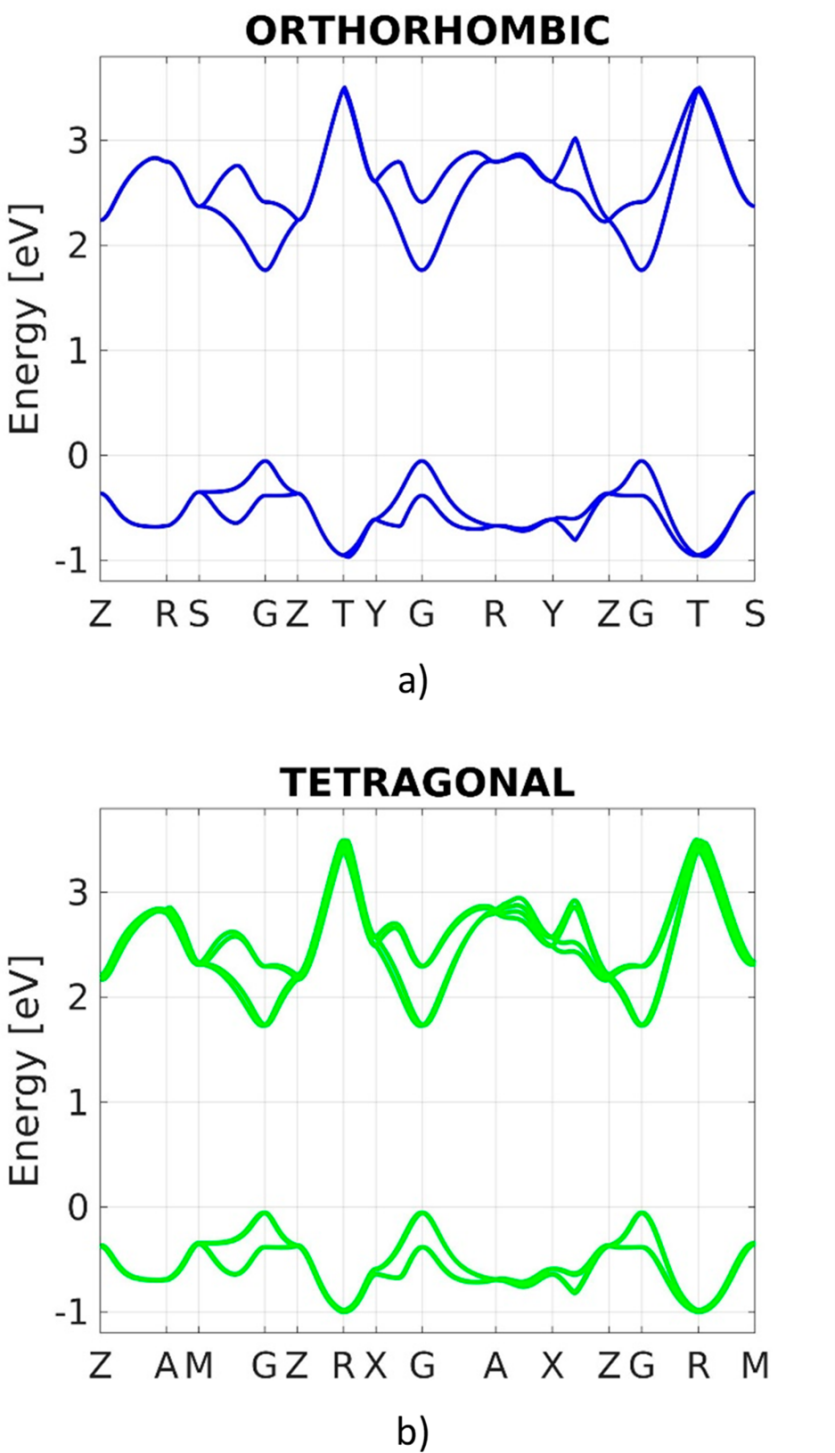
TB band structure of (a) orthorhombic and (b) tetragonal MAPbI_3_ derived after PSO.

**Table 1:** Energy Gap Values (eV) for Orthorhombic and Tetragonal MAPbI_3_

	orthorhombic	tetragonal
TB (after PSO)	1.82	1.78
DFT-GW^33^	1.81	1.67
experiment^33^	1.65	1.61

**Table 2:** Effective Masses for Holes and Electrons of Tetragonal MAPbI_3_ along the Directions Γ → *X* and Γ → *Z*

	TB (after PSO)	SOC-DFT^39^	SOC-GW^39^
*m*_*e*__,Γ→*X*_	0.1	0.16	0.17
*m*(_*h*,Γ→*X*_	0.13	0.22	0.20
*m*_*e*__,Γ→*Z*_	0.3	0.26	0.29
*m*_*e,Γ*__→*Z*_	0.4	0.44	0.40

Finally, the valence band offset between the two crystal structures is obtained by DFT calculations, following the steps described in the reference work of Weston et al.^41^ The position of the valence band maximum (VBM) with respect to the average electrostatic potential is determined from the DFT bulk calculations, separately for each crystal. Then, the alignment of the average electrostatic potential between the two crystals is determined from a supercell calculation, analyzing the average of the electrostatic potential in the bulk-like region of each crystal. Concerning the supercell calculation, we considered the *x*–*y* plane-averaged electrostatic potential integrated along the *z* direction over the length of the unit cell. The calculated data exhibit the superposition of a polarization potential, originating from the heterojunction between the two crystals, and a step-like potential, representing the electrostatic potential difference between the bulk-like regions of the two crystals. The computational details are reported in Section II of the Supporting Information. Combining the information from bulk calculations, i.e., the position of the VBM with respect to the mean electrostatic potential for the two separate bulk materials, with the electrostatic potential difference derived by superlattice calculations, we obtain the valence band alignment reported in Figure 2. The valence band offset between tetragonal and orthorhombic MAPbI_3_ is 0.011 eV, thus, the conduction band discontinuity is equal to 0.029 eV, i.e., the band gap difference between the two materials minus the valence band discontinuity value.

**Figure 2:**
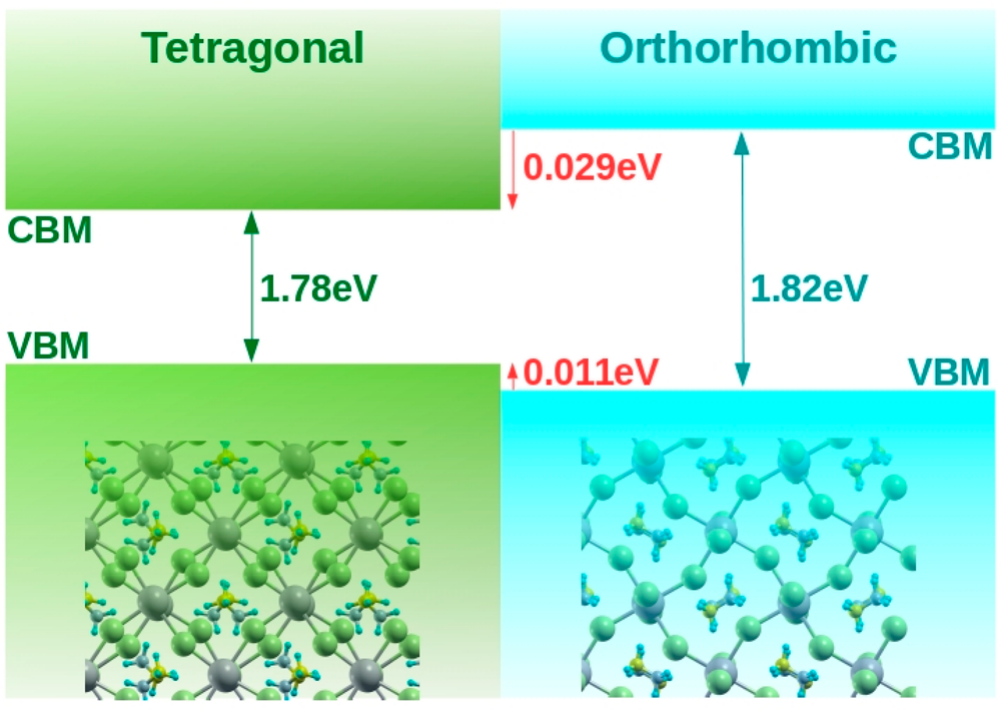
Band alignments between tetragonal and orthorhombic MAPbI_3_ derived by the combination of bulk and superlattice DFT calculations.^41^ The conduction band discontinuity is obtained considering the TB band gap values.

In Figure 3a, the conduction band (CB) and VB states of the system are reported for the considered tetragonal domain dimensions. We can see that the energy gap is significantly reduced for the 14 nm tetragonal domain system, as well as the value of the CB minimum (CBM), while the difference in VBM, CBM, and energy gap values between the 5 nm and the 9 nm tetragonal domain system is not relevant. On the other hand, the VBM value slightly increases for the largest domain size. In fact, the domain size dependence of the energy gap is almost equal to the CBM behavior. These trends originate from the spatially localized ground state electron wave function within the tetragonal domain. In fact, the l4 nm tetragonal segregation is sufficiently large to induce spatial localization of carriers. This phenomenon is clearly represented in panel b of Figure 3, where the isosurface containing the 50% of the total ground state density of the electron is depicted for the 14 nm tetragonal nanodomain. On the contrary, in panel c of Figure 3, where the same quantity is shown for the 5 nm tetragonal segregation, we can see that the spatial localization of the ground state electron wave function is not observed, even if the isosurface in panel c contains only the 10% of the total ground state density. Furthermore, as the conduction band discontinuity is much more pronounced than the valence band one, we do not expect a significant localization of the ground state hole wave function. In fact, the VBM value is almost unaffected by the dimension of the tetragonal segregation.

**Figure 3:**
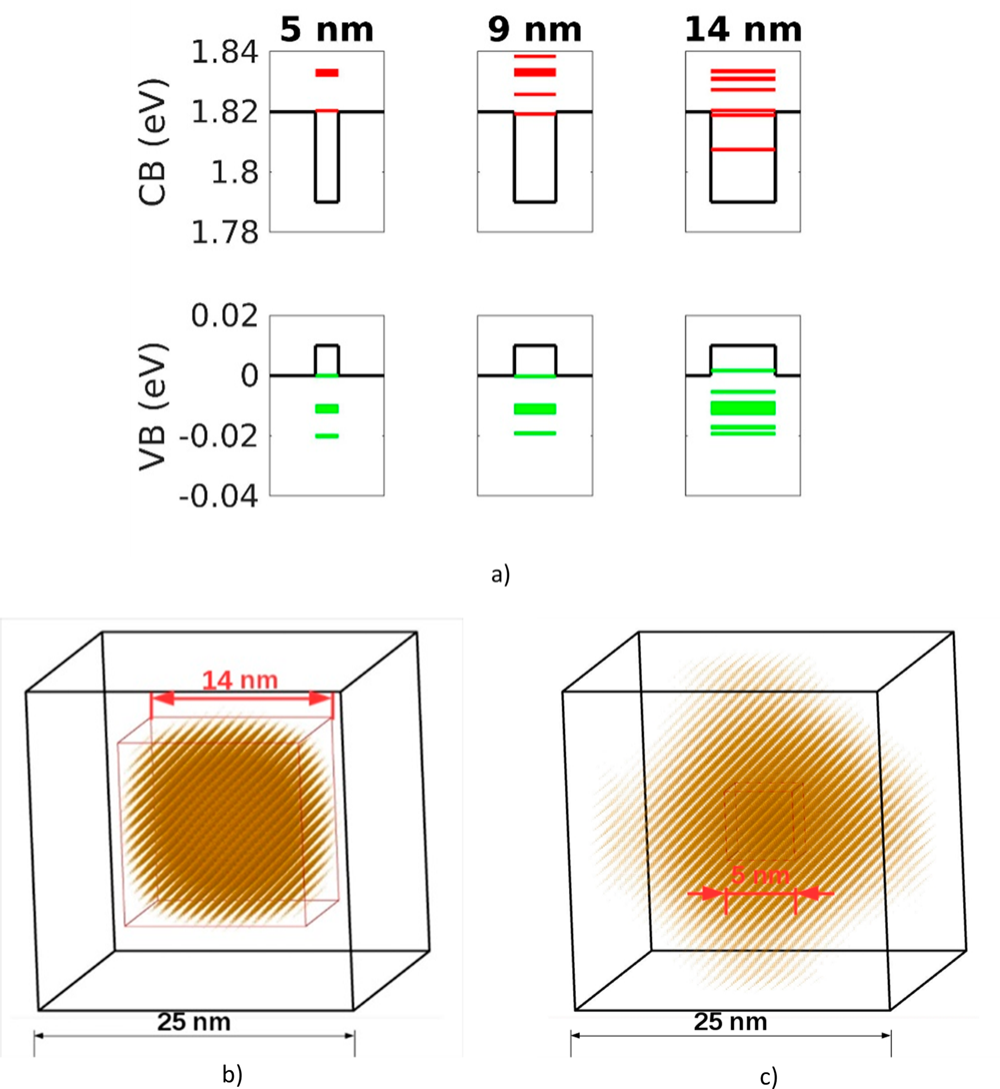
(a) Bulk band edge profiles across the considered tetragonal domains and energies of the valence (green) and conduction (red) band states at the gamma point. (b) Ground state electron wave function for the 14 nm tetragonal domain system. The isosurfaces containing 50% of the total ground state density are shown. (c) Ground state electron wave function for the 5 nm tetragonal domain system. The isosurfaces containing 10% of the total ground state density are shown.

In Figure 4, we show how the emission spectrum varies when the dimension of the tetragonal nanosegregation is increased from 5 to 14 nm. The three spectra are calculated at 150 K and normalized to unity. As expected from the behavior of the energy gap discussed above, we can see in the figure that, only for the 14 nm tetragonal segregation, the red-shift of the peak emission energy is observed. Furthermore, in the emission spectrum of the system with the highest dimension of the tetragonal domain, depicted by the cyan area plot in Figure 4, both the low-energy and the high-energy peaks, respectively associated with the tetragonal and the orthorhombic crystal structures, are visible. The low-energy feature in the calculated spectrum is due to the contribution of the electron ground state localized within the low-band gap tetragonal crystal, while the high-energy feature originates from higher-energy states coming from the prevalent orthorhombic phase. As discussed for Figure 3, the 5 nm and the 9 nm tetragonal segregations are not sufficiently large to yield a spatially localized electron ground state, thus, for these systems, the low-energy peak is not observed in the calculated spectra. The results obtained with different values of the band-offset are reported in Section III of the Supporting Information, for completeness.

**Figure 4:**
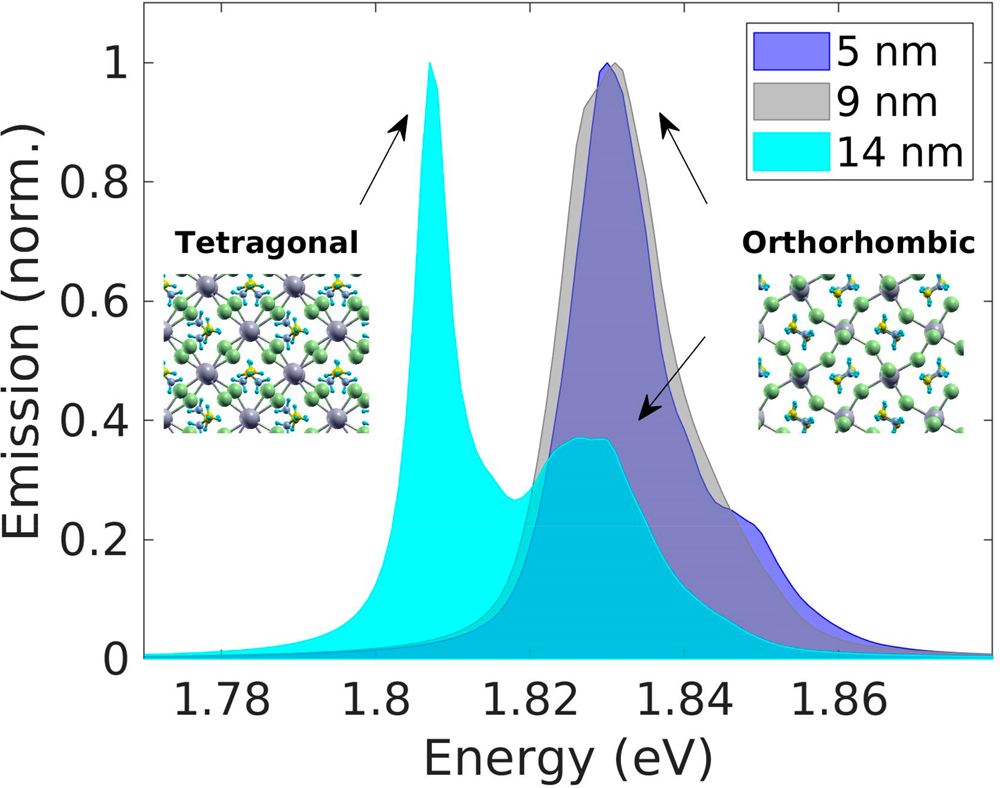
Emission spectra calculated at 150 K for the three considered tetragonal domain dimensions.

A consistent description of the emission properties of the system can be obtained as a superposition of the calculated spectra for the minimum and maximum tetragonal domain dimension, i.e., *xE*_5*nm*_ + (1 – *x*) *E*_14*nm*_, where *E*_5*nm*_ and *E*_14*nm*_ are the calculated emission spectra for the 5 and 14 nm domain size, respectively, and *x* is a temperature related weighting parameter derived by fitting the measured spectra. The results are represented in parts a–d of Figure 5, where panels a and c show the experimental data obtained by PL measurements in a temperature range going from 10 to 100 K with incident power density of 1.3 and 13 W/cm^2^, respectively, and the related fitted spectra are shown in panels b and d. In order to fit the experimental data, we have used a weighting parameter that is not a linear function of temperature. Thus, the emission properties of the system cannot be simply described assuming that the fraction of tetragonal phase decreases when the temperature is lowered. The behavior of the fitting parameter and the experimental setup for the PL measurements are discussed in Section IV of the Supporting Information. Note that the calculated spectra are blue-shifted with respect to the measured ones. This is due to the overestimation of the energy gap values, as discussed for Table 1. As it can be seen in Figure 5, the low energy feature, originating from localized states confined within the tetragonal domain, is visible even far below the transition temperature, indicating that a significant fraction of tetragonal MAPbI_3_ is still present.^27^ Some differences can be found between experimental and fitted spectra about the width and the temperature trend of tetragonal peak. In experimental data the tetragonal peak appears wider and less sharp, moreover seems to perform a slight red-shift lowering temperature below 100 K. These two features can be explained by the presence, in experimental data, of spectral components due to recombination on defect states lying within the band gap. Defect states related luminescence can appear in a wide spectral range and it tends to saturate increasing the temperature. Therefore, they can influence both the width and the position of other peaks.^27^ On the other hand, the red-shift of the orthorhombic peak observed in panels b and d is qualitatively consistent with the measurements of panels a and c. In fact, the occupation of the high-energy states associated with the orthorhombic crystal for different temperatures is accounted for in the theoretical spectra. The occupation decreases when the temperature is lowered, yielding an overall red-shift of the orthorhombic peak. This effect is further discussed in Section IV of the Supporting Information.

**Figure 5:**
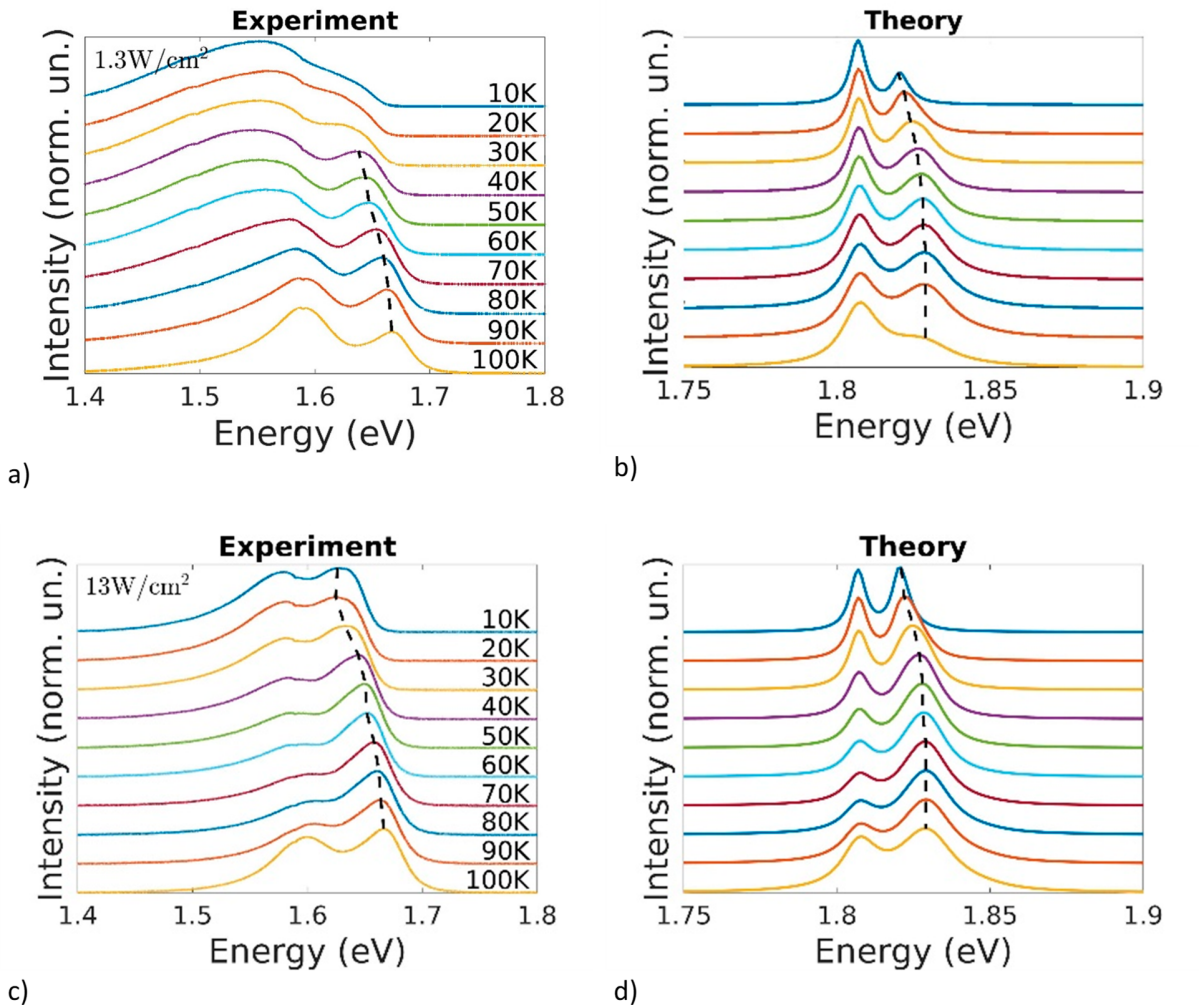
Experimental spectra obtained by PL measurements in a temperature range going from 10 to 100 K with incident power density of a 1.3W/cm^2^ and c 13W/cm^2^. The related theoretical spectra are shown in panels b and d, respectively. The higher density of excitation is taken into account using a carrier density ten times larger in panel d with respect to panel b.

In conclusion, our simulations demonstrate that the presence of residual nanodomains of tetragonal phase below the tetragonal-orthorhombic transition temperature can explain the low-energy features in the PL spectra of MAPbI_3_ perovskite down to 10 K. Even if our theoretical model does not address defect states recombination that can affect both the width and the position of PL peaks, the calculated spectra are consistent with the measured ones and the red-shift of the orthorhombic peak is confirmed. However, based on TB simulations and measurements, we think that the PL features cannot be quantitatively related to the exact fraction of residual tetragonal phase. In fact, the relative intensity of the tetragonal and orthorhombic peaks varies with the density of excitation employed in the experiment for a same value of temperature. Moreover, the fraction of tetragonal phase used in the theoretical spectra to fit the experimental trends is not a linear function of the temperature. Finally, the TB model, supported by PSO to determine the HME and by DFT calculations to estimate the VB offset, effectively capture the carriers’ localization phenomenon at the origin of the low-energy PL features.

## Supporting Information Available

Additional computational and experimental details, materials, and methods (PDF)
