# Supplementary material for: Role of Phase Nanosegregation in the Photoluminescence Spectra of Halide Perovskites
Source: J Phys Chem Lett. 2021 Nov 26;12(48):11659–65. doi: 10.1021/acs.jpclett.1c03378 (PMC8667165; doi:10.1021/acs.jpclett.1c03378)
Supplement: Supplementary file 1 — jz1c03378_si_001.pdf [file jz1c03378_si_001.pdf]

Supporting Information: The Role of Phase Nanosegregation in the Photoluminescence Spectra of Halide Perovskites

Alessia Di Vito,[*,†] Alessandro Pecchia,[‡] Matthias Auf der Maur,[†] Valerio Campanari,[†] Faustino Martelli,[¶] and Aldo Di Carlo[†,§]

†*University of Rome Tor Vergata, Via del Politecnico 1, 00133, Rome, Italy*

‡*CNR-ISMN, Via Salaria km 29,300, 00014 Monterotondo Stazione, Rome, Italy*

¶*CNR-IMM, Via del Fosso del Cavaliere, 00133, Rome, Italy*

§*LASE, Laboratory of Advanced Solar Energy, National University of Science and Technology "MISiS", Leninsky prospect 4, 119049, Moscow, Russia*

E-mail: alessia.di.vito@uniroma2.it

Tight binding (TB) parameterization through particle swarm optimization (PSO) algorithm

We employed the PSO algorithm[1] for the parameterization of the TB Hamiltonian matrix elements (HME) using the density functional theory (DFT) band dispersion as a target. Here, we show and discuss the results obtained for bulk $MAPbI_3$ perovskite. Note that the TB approach is not designed to yield the crystal structure, thus, an additional DFT based approach has been used to yield the atomic positions.

In PSO, a "swarm" of possible solutions, the "particles", to the problem under investigation moves within the search space of dimension D. Thus, each particle is treated as a point

in a D-dimensional space and explores the search area according to its own search experience and its companions' search experience. The performance of each particle is measured according to a predefined cost function f_{cost}, which is related to the problem to be solved. The ith particle is represented as $x_i = (x_{i,1}, ...x_{i,d}...x_{i,D})$. The best previous position (the position giving the minimum f_{cost} value) of the ith particle is recorded and represented as $p_i = (p_{i,1}, ...p_{i,d}...p_{i,D})$. The index of the best particle among all the particles in the population is represented by the symbol s. The rate of the position change (velocity) for particle i is represented as $v_i = (v_{i,1}...v_{i,d}...v_{i,D})$. The particles explore the search space according to the following equations:

$$v_{i,d} = a * v_{i,d} + b_1 * r_1(p_{i,d} - x_{i,d}) + b_2 * r_2(p_{s,d} - x_{i,d}) \tag{1}$$

$$x_{i,d} = x_{i,d} + v_{i,d} \tag{2}$$

where b_1 and b_2 are two positive constants, r_1 and r_2 are two random numbers in the range [0,1], and a is the inertia weight. Equation 1 is used to calculate the particle's new velocity according to its previous velocity (first term $a * v_{i,d}$) and the distances of its current position from its own best experience (second term $b_1 * r_1(p_{i,d} - x_{i,d})$) and the group's best experience (third term $b_2 * r_2(p_{s,d} - x_{i,d})$). Then the particle flies toward a new position according to equation 2. The inertia weight a is employed to control the balance between global (wide-ranging) and local (nearby) exploration abilities of the particles. A larger inertia weight facilitates global exploration (searching new areas) while a smaller inertia weight tends to facilitate local exploration to fine-tune the current search area.

Here, the $x_{i,d}$ positions represent the possible values of the TB HME, that determine the eigenvalues $\varepsilon_{n,k}^{TB}$ for the n band index at the k reciprocal space point. The target DFT eigenvalues $\varepsilon_{n,k}^{DFT}$ are calculated at the general gradient approximation (GGA) level, using full-relativistic projector-augmented-wave Perdew-Burke-Ernzerhof[2] pseudo-potentials. The

cost function is defined as

$$f_{cost} = \sum_{n,k} w_{n,k} (\varepsilon_{n,k}^{DFT} - \varepsilon_{n,k}^{TB})^2 \tag{3}$$

where $w_{n,k}$ are properly determined weights.

Of course, one could consider many other features in the target data set, and thus in the cost function definition, such as the band gap and effective masses values, determined by experimental measurements or state-of-art ab-initio calculations. Here, the experimental band gap value has been taken into account as target feature, adjusting the DFT energy gap to the measured one.

The optimization of the TB parameters is performed taking into account the band structure of all the three $MAPbI_3$ phases simultaneously. The target band structure of the material for each phase is calculated at the DFT-GGA level of approximation using the QuantumEspresso package,[3] adjusting the band gap energy to its experimental value.[4] The TB calculations are performed employing the TiberCAD software.[5] The choice of TB basis can be the minimal sp^3 set, as demonstrated by Boyer-Richard et al.[6] Moreover, the TB model does not include the MA cation, since it has been shown that the organic cation does not affect the band structure and density of states in these materials.[6] Thus, for each Pb and I atom, we consider one s and three p orbitals. No basis function are considered for any of the MA cations, consistently with DFT studies of the electronic states around the band gap that do not reveal significant weight stemming from MA.[7] We have 16 TB parameters related to the Pb-I inorganic cage, namely: two spin-orbit coupling (SOC) parameters (Pb SOC and I SOC), four diagonal HME related to the atomic energies of the chemical constituents Pb and I (Pb on-site 's', Pb on-site 'p', I on-site 's' and I on-site 'p'), five transfer HME refered to the overlap integral of first neighbor atomic functions (Pb 's'/I 's' σ, Pb 's'/I 'p' σ, Pb 'p'/I 's' σ, Pb 'p'/I 'p' σ and Pb 'p'/I 'p' π) and five scaling parameters. The latters are related to the transfer HME by the following relation:

$$HME_i^{tran} = HME_{i0}^{tran}(d_0/d)^{\alpha_i} \quad (4)$$

where HME_{i0}^{tran} and d_0 are respectively the transfer HME and the interatomic distance without strain, and α_i is the scaling parameter. Thus, Equation 4 accounts for the influence of the strain, using a simple variation of the five transfer HME as a function of the interatomic distance d.

First of all, we found the optimal values of the algorithm parameters a, b_1 and b_2. The cost function values obtained for different sets of the algorithm parameters a, b_1 and b_2 are reported in Table S1 and represented in Figure S1, for clarity. It can be seen that the f_{cost} value decreases when a is increased, since the global searching is facilitated and the particles explore new areas in the search space. Moreover, when the collaboration among particles is aided (i.e., when the value of b_2 goes up) the f_{cost} value goes down. The best result is obtained for the parameters set $a = 0.5$, $b_1 = 1$, $b_2 = 1.5$.

Table S1: Cost function values obtained for different sets of the algorithm parameters a, b_1 and b_2. In all cases, the optimization is performed using 70 particles and the maximum number of PSO iterations is set to 100.

a	b_1	b_2	f_{cost}
0.1	1	1	0.39
0.3	1	1	0.13
0.5	1	1	0.035
0.5	0.5	1	0.033
0.5	1.5	1	0.034
0.5	1	0.5	0.043
0.5	1	1.5	0.031

The results obtained for the 16 TB parameters with the PSO algorithm are reported in Tables S2, S3, S4 and S5, and compared, where available, with the values found in the study of Boyer-Richard et al.[6] with an empirical fitting procedure. The convergence for each TB

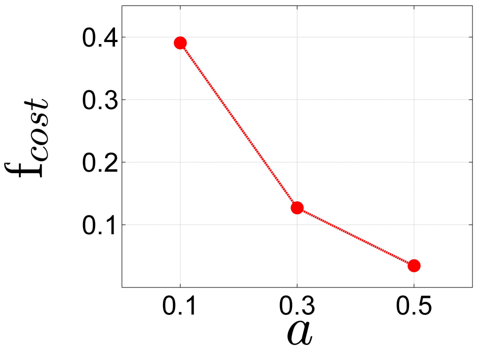

(a)

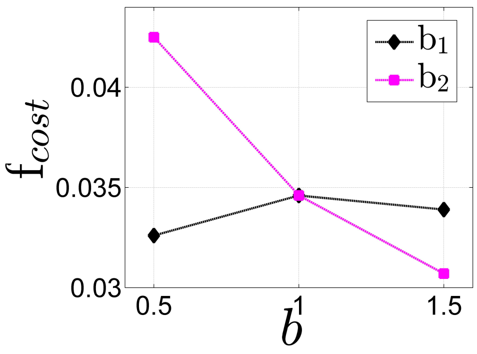

(b)

Figure S1: Cost function values obtained for different sets of the algorithm parameters a, b_1 and b_2. In panel (a), the parameters b_1 and b_2 are set to 1. In panel (b), the parameter a is set to 0.5. In all cases, the optimization is performed using 70 particles and the maximum number of PSO iterations is set to 100.

parameter value versus the number of PSO iterations is also shown in the Tables. It can be seen that the choice of the number of particles (i.e., 70 particles) and of maximum iterations (i.e., 100 iterations) is sufficient to have a converged value for all the TB parameters, in this case.

The optimized TB band structure is shown in Figure S2 and compared with the target DFT band structure for each phase of $MAPbI_3$. Moreover, in Table S6, we report the energy gap values obtained with the TB model after PSO. These are compared with the measurements and the state-of-the-art GW calculations presented by Quarti et al.[4]

Valence band offset calculation from density functional theory

The DFT calculation of the valence band offset between tetragonal and orthorhombic $MAPbI_3$ is based on the reference study of Weston *et al.*[8] First, separate calculations for each bulk material are performed in which the valence band maximum (VBM) is determined with respect to the average electrostatic potential in the respective material. But this is not sufficient, since the average electrostatic potential is not defined on an absolute energy scale. The bulk

Table S2: Spin-orbit coupling parameters obtained with PSO. The optimization is performed using $a = 0.5$, $b_1 = 1$, $b_2 = 1.5$, 70 particles and the maximum number of PSO iterations is set to 100. The PSO results are compared with the empirical parameterization of Boyer-Richard et al.[6] Moreover, the convergence of each parameter value versus the number of PSO iterations is shown.

	PSO	Ref.[6]	convergence
Pb SOC	0.4405	0.4333	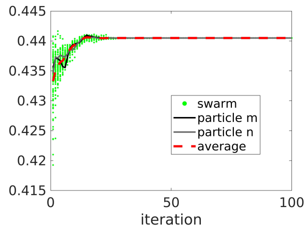
I SOC	0.29946	0.3	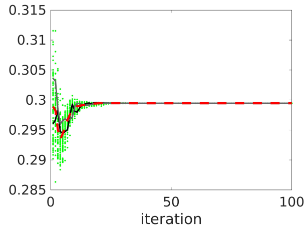

calculations need to be complemented by interface calculations to determine the alignment of the average electrostatic potential between the to materials. This is done by performing a calculation for a superlattice and examining the average of the electrostatic potential in the bulk-like region of each layer. Combining the information from bulk calculations with the electrostatic potential difference then produces the valence band alignment. Note that the lattice parameters of the orthorhombic phase are fixed to that of the tetragonal phase. Thus, the bulk calculation to determine the VBM of the orthorhombic phase is performed for the strained system.

DFT simulations are performed at the GGA level of approximation using the Quantum Espresso package.[3] We employed scalar-relativistic PBE pseudopotentials.[2] The energy cut-off for the plane wave basis set is set to 40 Ry and we used a 6×6×1 k-point grid for reciprocal space integration. The supercell structure for the calculation of the electrostatic potential difference between tetragonal and orthorhombic $MAPbI_3$ is shown in Figure S3. Note that

the MA cations are aligned along the x direction, while the c crystal axis is aligned along the z direction.

In Figure S4, we show the calculated electrostatic potential, V_{el}, for a superlattice with four tetragonal and four orthorhombic $MAPbI_3$ unit cells. The c crystal axis is aligned along the z direction. The black and red solid lines represent the $x - y$ plane-averaged and the mean electrostatic potential, respectively. The green solid line represents the $x - y$ plane-averaged electrostatic potential integrated along the z direction over the length of the unit cell. This exhibits the linear behavior expected for a polarization potential generated by the heterojunction between the two materials. The linear interpolation of the calculated data is derived in the bulk-like region of the two materials and is depicted in Figure S4 by the magenta dashed line.

In Figure S5, only the mean electrostatic potential and the linear interpolation of the calculated data are represented by the red and magenta solid lines, respectively, for the 4+4 superlattice. The superposition of the polarization potential (light gray dashed line), originating from the heterojunction between the two crystals, and the step-like potential (dark gray dashed line), representing the electrostatic potential difference between the bulk-like regions of the two crystals, is shown.

Band-offset dependence of the system's emission properties

For completeness, we show here the results derived for several values of the valence band-offset between tetragonal and orthorhombic $MAPbI_3$. Namely, in Figure S6 and S7 the considered valence band discontinuity is 0.04eV, Figure S8 and S9 are obtained for a valence band discontinuity of 0.02eV, while in Figure S10 and S11 a conduction band discontinuity of 0.04eV is addressed.

Experimental conditions for photoluminescence measurements and fitting parameters

The photoluminescence measurements were performed by exciting the samples with a 405nm CW laser diode focused to a diameter of approximately 100μm. The sample was held at temperatures in the range 10-100K in a close-loop He cryostat. The luminescence was detected with a Peltier-cooled CCD camera after having been dispersed in a 30cm long monochromator with a 1200grooves/mm grating. The measurement was performed every 10K heating the sample from 10K to 100K.

In Figure S12a, the experimental spectra obtained by PL measurements in a temperature range going from 10 to 100K with incident power density of 255W/cm^2 are shown. From the theoretical viewpoint, a consistent description of the emission properties of the system can be obtained as a superposition of the calculated spectra for the minimum and maximum tetragonal domain dimension, i.e. $x(E_{5nm}) + (1 - x)(E_{14nm})$, where E_{5nm} and E_{14nm} are the calculated emission spectra for the 5nm and 14nm domain size, respectively, and x is a weighting parameter derived by fitting the measured spectra. In Figure S12b, the theoretical spectra related to the 255W/cm^2 excitation density measurements are shown.

In Figure S12c-e, the value of x as a function of temperature is reported. Panels c), d) and e) refer to the spectra obtained with incident power density of 1.3W/cm^2, 13W/cm^2 and 255W/cm^2, respectively. Note that, for a same value of temperature, the value of x varies whit the density of excitation employed in the experiment. Moreover, the weighting parameter is not a linear function of the temperature.

In Figure S13, the emission spectra calculated at 10K and 50K for the 5nm and the 14nm tetragonal domain dimension are reported. Here, the spectra are not normalized to unity. It can be seen that, decreasing the temperature from 50K to 10K, the high-energy peak results red-shifted and its intensity increases. In fact, lowering the temperature, the high-energy states that contribute to the orthorhombic peak are less occupied, and the spectral feature

is essentially related to the ground state optical transition. The momentum matrix element of the ground state optical transition for the 5nm domain dimension is significantly higher than the others and the transition energy between the electron ground state and the hole ground state is 1.82eV. This is shown in Figure S14, where the momentum matrix elements associated to the optical transitions from the first three electron states to the first three hole states are reported as a function of the related transition energy. Thus, the emission spectrum calculated at 10K for the 5nm domain dimension exhibits a peak at 1.82eV with higher intensity with respect to the others.

Finally, we report in Figure S15 the results of the degradation control experiment. The normalized PL spectra measured at 10K are shown, where the low-energy and high-energy PL features are labeled by LE and HE, respectively. The sample was excited with 26mW/cm^2 (orange), then the power density was switched to 380mW/cm^2 (green) and finally again to 26mW/cm^2 (blue). The superposition of the orange and blue solid lines demonstrates that the spectrum variation induced by high power densities (with promotion of LE component) is a reversible process and that it is not consequence of material degradation.

References

(1) Kennedy, J.; Eberhart, R. Particle swarm optimization. Proceedings of ICNN'95 - International Conference on Neural Networks. 1995; pp 1942–1948 vol.4.

(2) Perdew, J. P.; Burke, K.; Ernzerhof, M. Generalized gradient approximation made simple. *Physical Review Letters* **1996**, *77*, 3865.

(3) Giannozzi, P.; Baroni, S.; Bonini, N.; Calandra, M.; Car, R.; Cavazzoni, C.; Ceresoli, D.; Chiarotti, G. L.; Cococcioni, M.; Dabo, I., et al. QUANTUM ESPRESSO: a modular and open-source software project for quantum simulations of materials. *Journal of Physics: Condensed Matter* **2009**, *21*, 395502.

(4) Quarti, C.; Mosconi, E.; Ball, J. M.; D'Innocenzo, V.; Tao, C.; Pathak, S.; Snaith, H. J.; Petrozza, A.; De Angelis, F. Structural and optical properties of methylammonium lead iodide across the tetragonal to cubic phase transition: implications for perovskite solar cells. *Energy & Environmental Science* **2016**, *9*, 155–163.

(5) TiberCAD simulation package. http://www.tibercad.org.

(6) Boyer-Richard, S.; Katan, C.; Traore, B.; Scholz, R.; Jancu, J.-M.; Even, J. Symmetry-based tight binding modeling of halide perovskite semiconductors. *The Journal of Physical Chemistry Letters* **2016**, *7*, 3833–3840.

(7) Even, J.; Pedesseau, L.; Jancu, J.-M.; Katan, C. Importance of spin–orbit coupling in hybrid organic/inorganic perovskites for photovoltaic applications. *The Journal of Physical Chemistry Letters* **2013**, *4*, 2999–3005.

(8) Weston, L.; Tailor, H.; Krishnaswamy, K.; Bjaalie, L.; Van de Walle, C. Accurate and efficient band-offset calculations from density functional theory. *Computational Materials Science* **2018**, *151*, 174–180.

Table S3: On-site parameters obtained with PSO. The optimization is performed using $a = 0.5$, $b_1 = 1$, $b_2 = 1.5$, 70 particles and the maximum number of PSO iterations is set to 100. The PSO results are compared with the empirical parameterization of Boyer-Richard et al.[6] Moreover, the convergence of each parameter value versus the number of PSO iterations is shown.

	PSO	Ref.[6]	convergence
Pb on-site 's'	-9.1978	-9.01	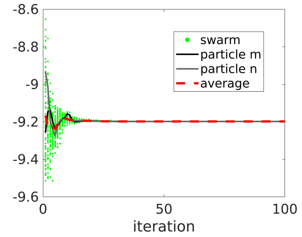
Pb on-site 'p'	2.2747	2.34	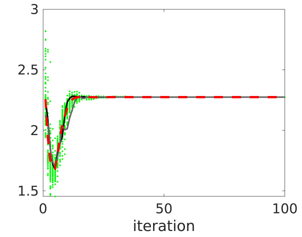
I on-site 's'	-12.8286	-13.01	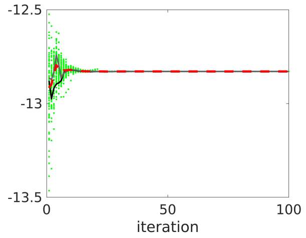
I on-site 'p'	-1.4723	-1.96	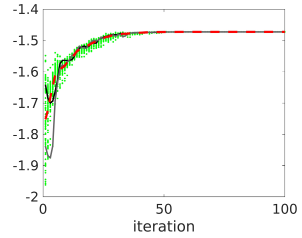

Table S4: Hopping parameters obtained with PSO. The optimization is performed using $a = 0.5$, $b_1 = 1$, $b_2 = 1.5$, 70 particles and the maximum number of PSO iterations is set to 100. The PSO results are compared with the empirical parameterization of Boyer-Richard et al.[6] Moreover, the convergence of each parameter value versus the number of PSO iterations is shown.

	PSO	Ref.[6]	convergence
Pb 's'/I 's' σ	-1.2097	-1.10	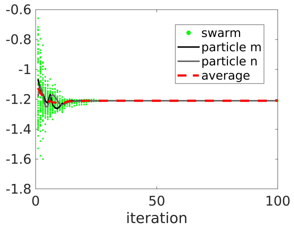
Pb 's'/I 'p' σ	1.0296	1.19	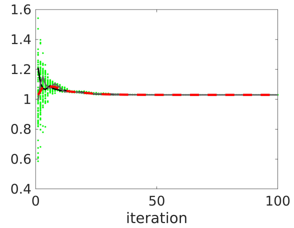
Pb 'p'/I 's' σ	0.65051	0.70	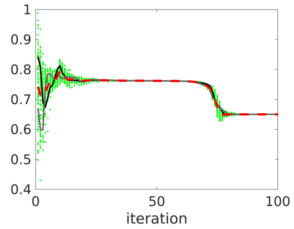
Pb 'p'/I 'p' σ	-2.034	-3.65	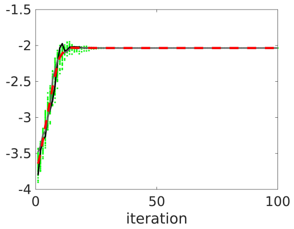
Pb 'p'/I 'p' π	0.55919	0.55	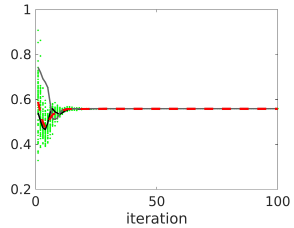

Table S5: Scaling parameters obtained with PSO. The optimization is performed using $a = 0.5$, $b_1 = 1$, $b_2 = 1.5$, 70 particles and the maximum number of PSO iterations is set to 100. The convergence of each parameter value versus the number of PSO iterations is shown.

	PSO	convergence
Pb ’s’/I ’s’ σ, scaling	1.7211	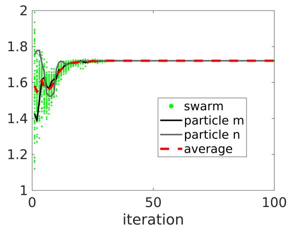
Pb ’s’/I ’p’ σ, scaling	1.4515	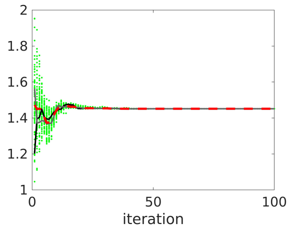
Pb ’p’/I ’s’ σ, scaling	1.4849	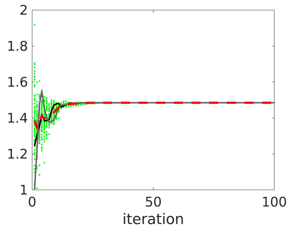
Pb ’p’/I ’p’ σ, scaling	1.5389	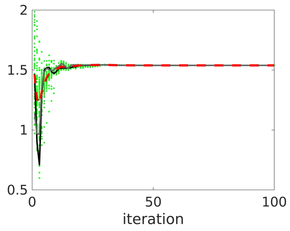
Pb ’p’/I ’p’ π, scaling	1.3782	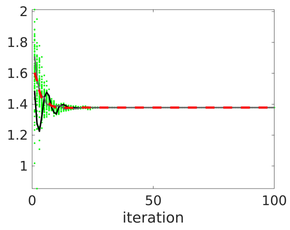

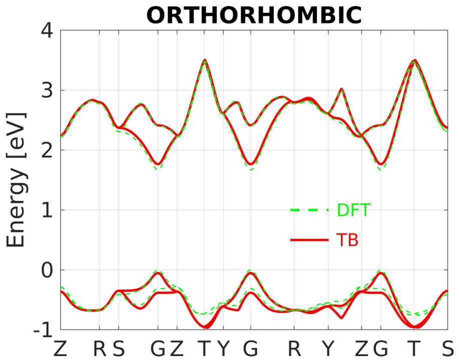

(a)

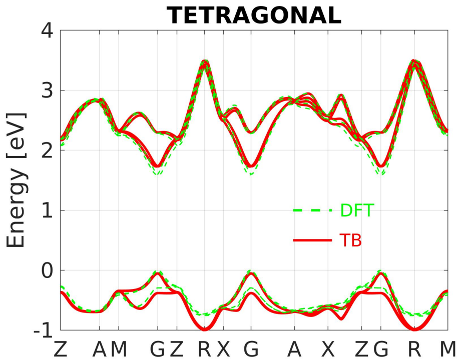

(b)

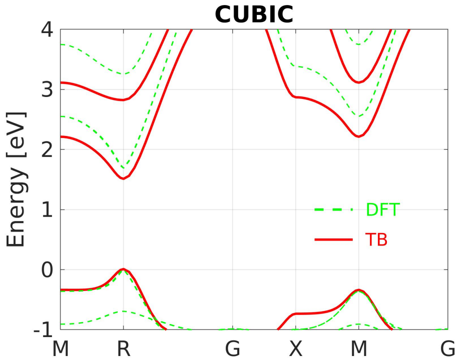

(c)

Figure S2: TB band structure of a) orthorhombic, b) tetragonal and c) cubic $MAPbI_3$ (red solid lines) derived after PSO and compared with the target DFT band structure (green dashed lines).

Table S6: Energy gap values (eV) obtained with TB after PSO for orthorhombic, tetragonal and cubic $MAPbI_3$. The results are compared with the experimental and theoretical values obtained by Quarti et al.[4]

	orthorhombic	tetragonal	cubic
TB (after PSO)	1.82	1.78	1.51
DFT-GW[4]	1.81	1.67	1.28
Experiment[4]	1.65	1.61	1.69

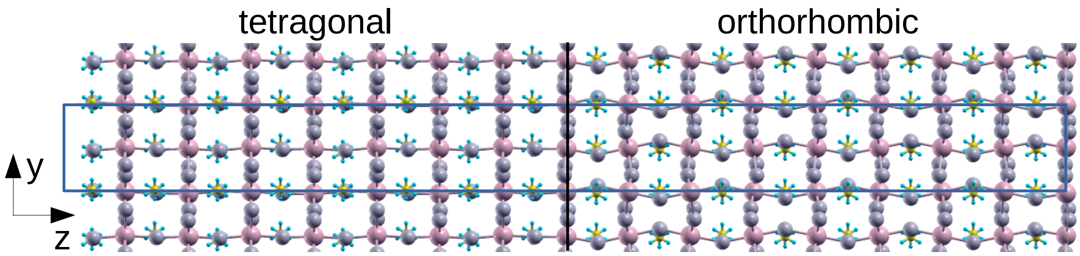

Figure S3: Supercell structure for the DFT calculation of the electrostatic potential difference between tetragonal and orthorhombic $MAPbI_3$. The c crystal axis is aligned along the z direction and the MA cations are aligned along the x direction. The translational asymmetric unit of repetition is represented by the blue solid line.

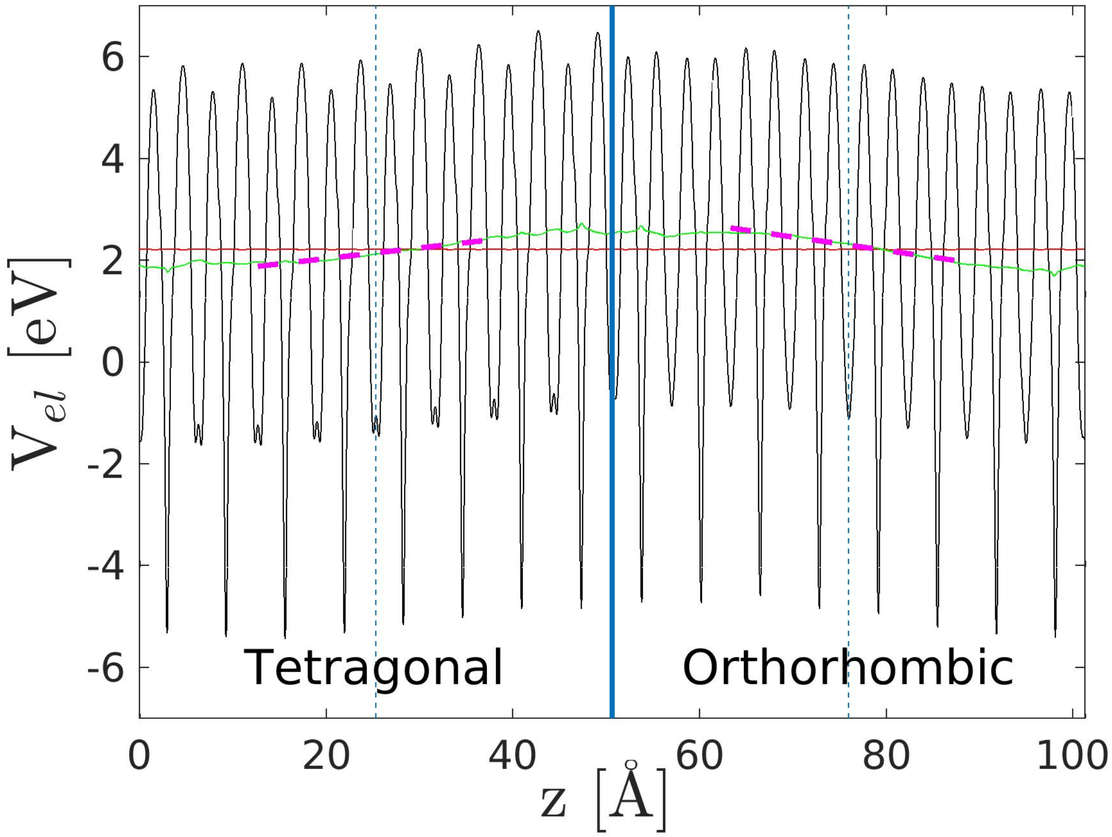

Figure S4: DFT calculated electrostatic potential for a superlattice with four tetragonal and four orthorhombic $MAPbI_3$ unit cells. The c crystal axis is aligned along the z direction. The black and red solid lines represent the $x - y$ plane-averaged and the mean electrostatic potential, respectively. The green solid line represents the $x - y$ plane-averaged electrostatic potential integrated along the z direction over the length of the unit cell. The linear interpolation of the calculated data in the bulk-like region of the two materials is depicted by the magenta dashed line.

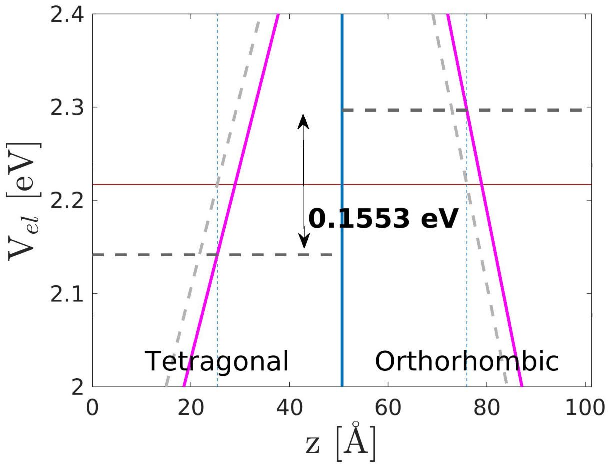

Figure S5: Electrostatic potential for a superlattice with four tetragonal and four orthorhombic $MAPbI_3$ unit cells. The c crystal axis is aligned along the z direction. The red solid line represents the mean electrostatic potential. The magenta solid line represents the linear interpolation of the $x - y$ plane-averaged electrostatic potential integrated along the z direction over the length of the unit cell. The electrostatic potential difference between tetragonal and orthorhombic $MAPbI_3$ is depicted by the dark gray dashed line, while the light gray dashed line represents the polarization potential.

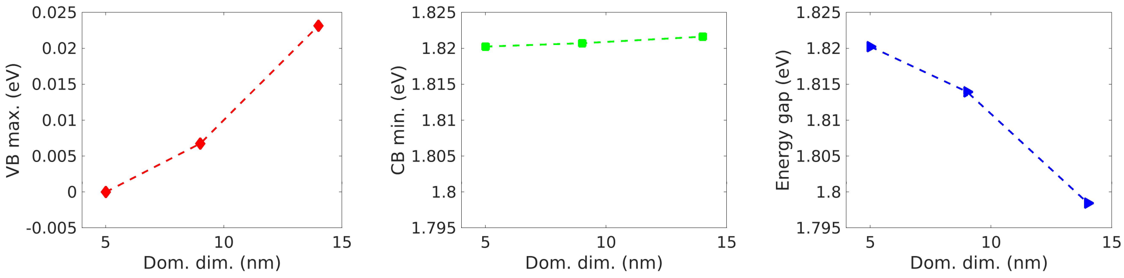

(a) (b) (c)

Figure S6: a) Valence band maximum, b) conduction band minimum and c) energy gap values as a function of the tetragonal domain dimension for a valence band discontinuity of 0.04eV.

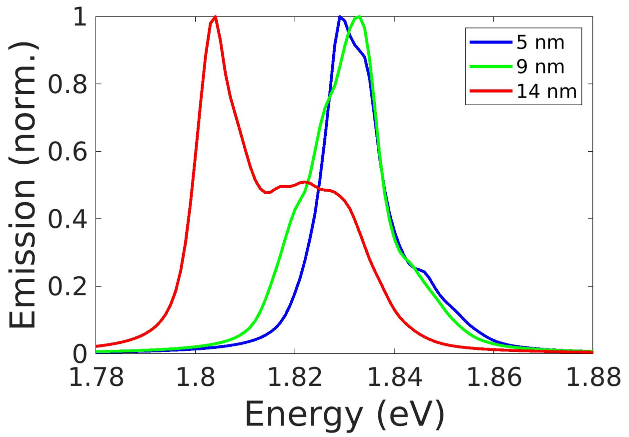

Figure S7: Emission spectra calculated at 150K for the three considered tetragonal domain dimensions. The valence band discontinuity is 0.04eV.

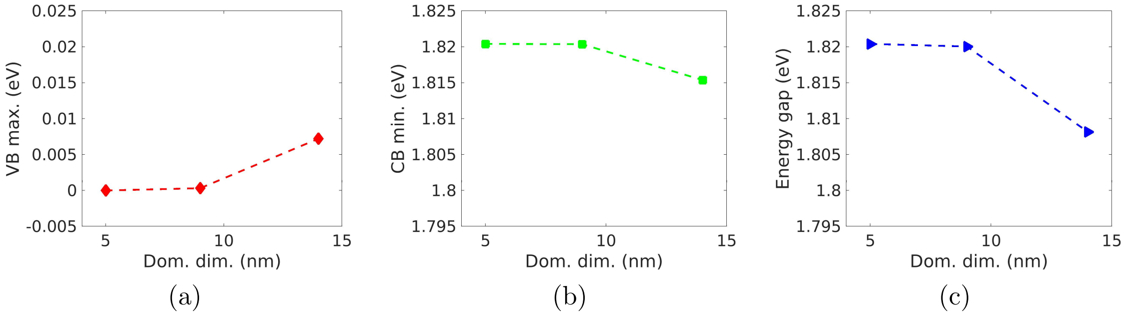

Figure S8: a) Valence band maximum, b) conduction band minimum and c) energy gap values as a function of the tetragonal domain dimension for a valence band discontinuity of 0.02eV.

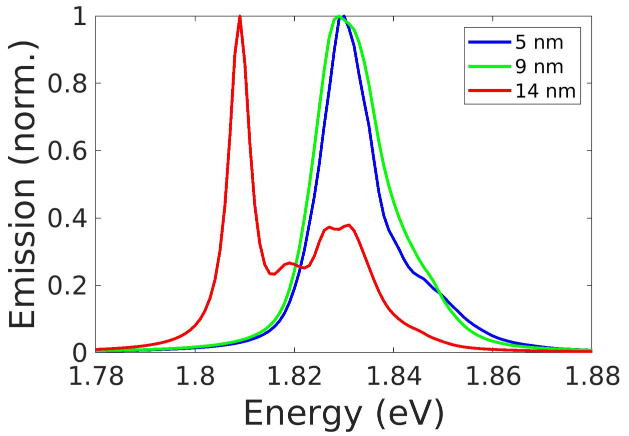

Figure S9: Emission spectra calculated at 150K for the three considered tetragonal domain dimensions. The valence band discontinuity is 0.02eV.

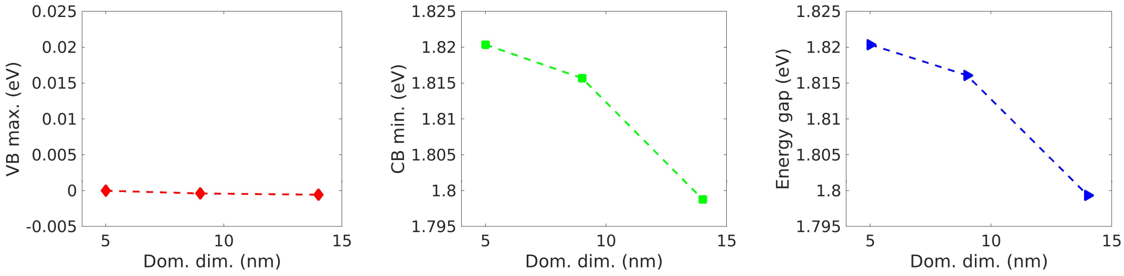

(a) (b) (c)

Figure S10: a) Valence band maximum, b) conduction band minimum and c) energy gap values as a function of the tetragonal domain dimension for a conduction band discontinuity of 0.04eV.

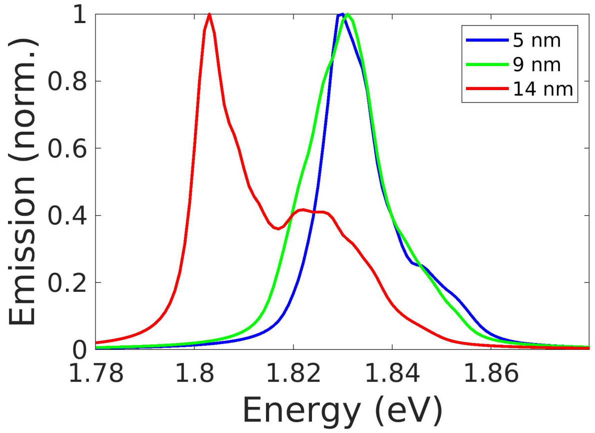

Figure S11: Emission spectra calculated at 150K for the three considered tetragonal domain dimensions. The conduction band discontinuity is 0.04eV.

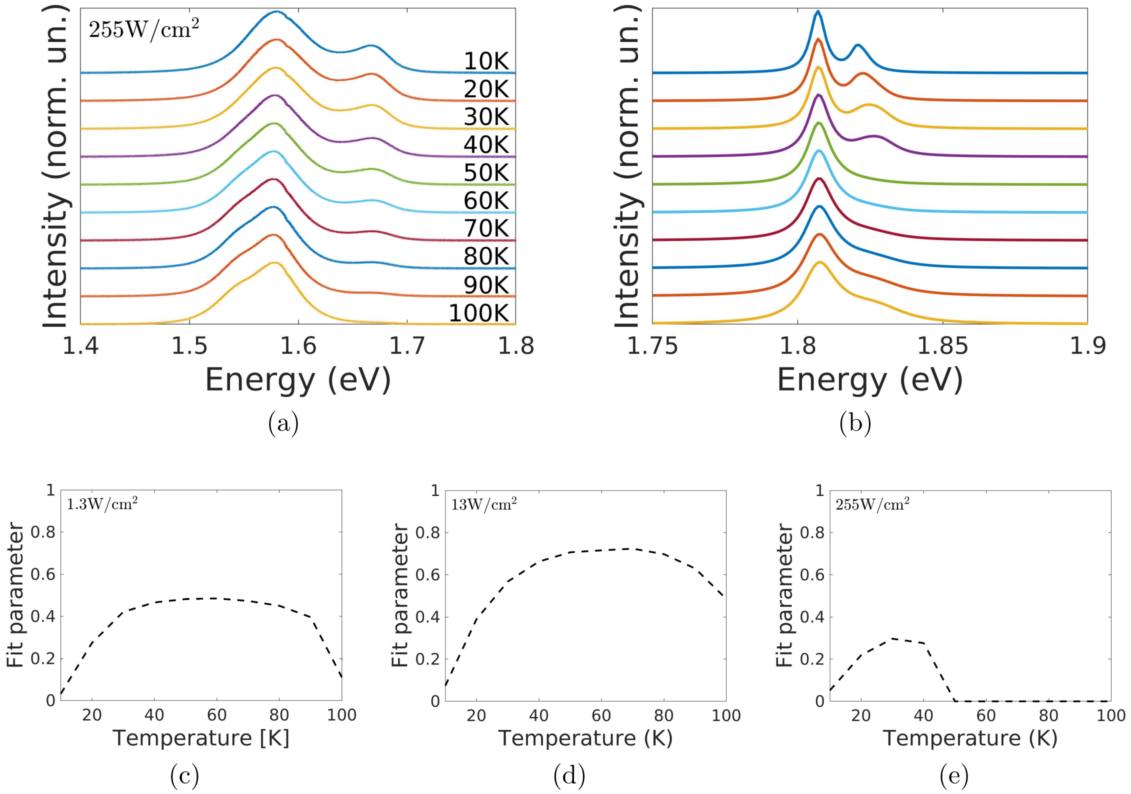

Figure S12: a) Experimental spectra obtained by PL measurements in a temperature range going from 10 to 100K with incident power density of 255W/cm^2. The related theoretical spectra are shown in panel b). Weighting parameter x, used to fit the experimental photoluminescence spectra, as a function of temperature. Panels c), d) and e) refer to the spectra obtained with incident power density of 1.3W/cm^2, 13W/cm^2 and 255W/cm^2, respectively.

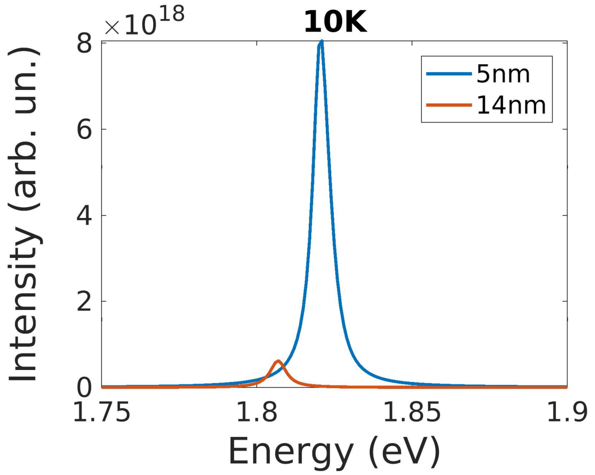

(a)

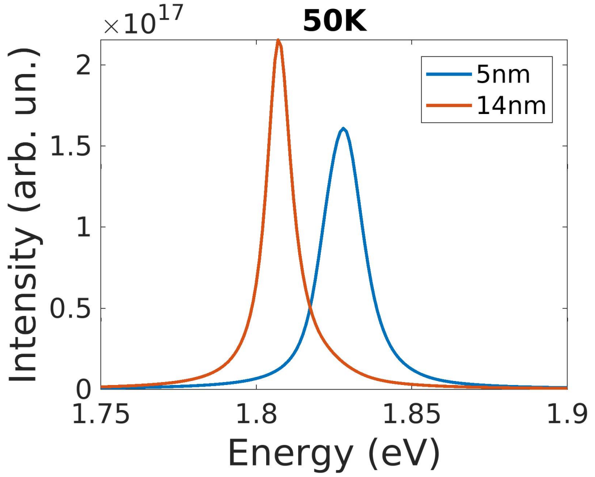

(b)

Figure S13: Emission spectra calculated at a) 10K and b) 50K for the 5nm and the 14nm tetragonal domain dimension.

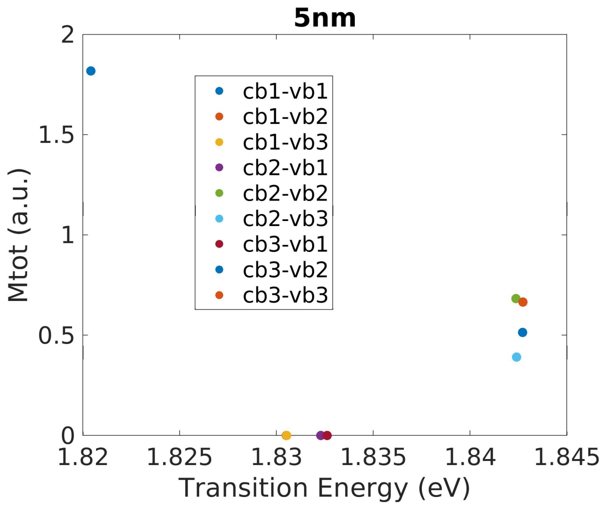

(a)

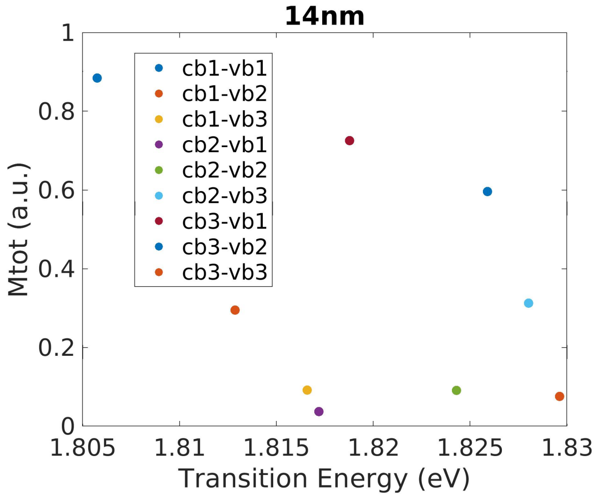

(b)

Figure S14: Momentum matrix elements of the optical transitions from the first three electron states to the first three hole states for a) the 5nm and b) the 14nm tetragonal domain dimension.

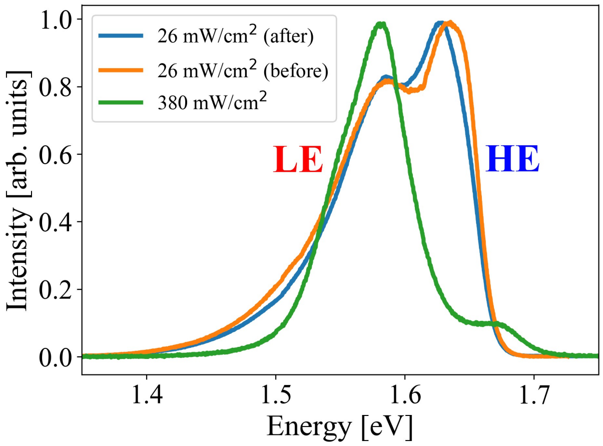

Figure S15: Normalized PL spectra at 10K for degradation control experiment. The sample was excited with 26mW/cm^2 (orange), then the power density was switched to 380mW/cm^2 (green) and finally again to 26mW/cm^2 (blue). The spectrum variation induced by high power densities (with promotion of LE component) is a reversible process and it is not consequence of material degradation.
